# Assessing infection control practices to protect health care workers and patients in Malawi from nosocomial transmission of Mycobacterium tuberculosis

**DOI:** 10.1371/journal.pone.0189140

**Published:** 2017-12-06

**Authors:** Robert J. Flick, Adamson Munthali, Katherine Simon, Mina Hosseinipour, Maria H. Kim, Lameck Mlauzi, Peter N. Kazembe, Saeed Ahmed

**Affiliations:** 1 Baylor College of Medicine Children’s Foundation Malawi, Lilongwe, Malawi; 2 University of North Carolina Project-Malawi, Lilongwe, Malawi; 3 University of Colorado School of Medicine, Denver, United States of America; 4 Baylor International Pediatric AIDS Initiative at Texas Children’s Hospital, Baylor College of Medicine, Houston, United States of America; 5 University of North Carolina at Chapel Hill, Chapel Hill, United States of America; 6 Malawi Ministry of Health National Tuberculosis Control Programme, Lilongwe, Malawi; University of New South Wales, AUSTRALIA

## Abstract

Transmission of *Mycobacterium tuberculosis* (TB) in health settings threatens health care workers and people living with HIV in sub-Saharan Africa. Nosocomial transmission is reduced with implementation of infection control (IC) guidelines. The objective of this study is to describe implementation of TB IC measures in Malawi. We conducted a cross-sectional study utilizing anonymous health worker questionnaires, semi-structured interviews with facility managers, and direct observations at 17 facilities in central Malawi. Of 592 health care workers surveyed, 34% reported that all patients entering the facility were screened for cough and only 8% correctly named the four most common signs and symptoms of TB in adults. Of 33 managers interviewed, 7 (21%) and 1 (3%) provided the correct TB screening questions for use in adults and children, respectively. Of 592 health workers, only 2.4% had been screened for TB in the previous year. Most (90%) reported knowing their HIV status, 53% were tested at their facility of employment, and half reported they would feel comfortable receiving ART or TB treatment at their facility of employment. We conclude that screening is infrequently conducted and knowledge gaps may undercut its effectiveness. Further, health care workers do not routinely access TB and HIV diagnostic and treatment services at their facility of employment.

## Introduction

Nosocomial transmission of *Mycobacterium tuberculosis* (TB) is a major public health threat in sub-Saharan Africa (SSA) [1,2]. Health care workers (HCWs), who face frequent occupational exposure, are at particularly high risk [3–7]. People living with HIV (PLHIV) are also at risk, due to impaired immune function and increased exposure to congregate health settings [8–10]. Numerous nosocomial outbreaks have been documented these two groups [11,12].

Nosocomial exposure and transmission of TB is reduced with implementation of infection control (IC) guidelines that promote the rapid identification, triage, and isolation of potentially infectious patients, the improvement of ventilation, and the availability of personal protective equipment [13]. Routine TB screening for HCWs, combined with access to confidential HIV testing and antiretroviral therapy is crucial to both protect the health of providers and prevent HCWs from becoming sources of TB transmission [9,10,14].

The little available evidence describing the extent of IC guideline implementation in SSA suggests HCWs have limited access to confidential HIV testing, with both lack of services and a perceived lack of confidentiality contributing to low testing uptake [15–17]. As the region adopts WHO guidelines for universal ART, it is imperative to better understand the state of IC at facilities tasked with absorbing the influx of ART patients. Should IC be lacking, investments in health systems strengthening will be critical to ensure that accessing ART does not expose new patients to avoidable risk.

Malawi is a landlocked country in SSA that is implementing universal ART for its estimated million PLHIV [18]. Prior to the introduction of ART, TB was responsible for almost half of recorded deaths in HCWs [3]. Guidelines for IC have been developed, but their routine practice has not been characterized. In this study, we aim to describe the implementation of IC guidelines prior to the shift to universal ART in Malawi.

## Methods

### Study design and setting

Malawi is a resource-limited country of 16 million people in sub-Saharan Africa, with an adult HIV prevalence of 10.3% and a TB incidence rate among adults of 227 cases per 100,000 [19]. We conducted a cross-sectional assessment in seventeen Malawi Ministry of Health (MOH) operated facilities (six hospitals, eleven health centers) in four districts in Malawi’s central region with a total estimated catchment area population of 1.2 million people. Sites were visited between September 2014 and July 2015. Site selection was deliberate and made in coordination with the MOH, with all surveyed facilities supported in various degrees by the Tingathe community health worker-based outreach program [20,21].

At the time of the study, NTP guidelines recommended universal cough screening for everyone entering health facilities. Intensified case finding was recommended for PLHIV according to WHO guidelines that recommended asking about a history of fever, cough, weight loss (poor weight gain in children), or night sweats. Guardians of children were also asked about a home TB contact. Presumptive TB patients were evaluated by sputum smear microscopy, and chest radiography if necessary. The GeneXpert MTB/RIF Assay was used for critically ill patients, or PLHIV with a negative smear result.

### IC guidelines assessed

We assessed guidelines according to the four hierarchies widely used by both the WHO and the Malawi NTP: managerial activities, administrative controls, environmental controls, and personal protective equipment [22,23]. We included HCW access to care as a separate fifth category (Box 1). Three approaches were used to assess guideline implementation: i.) an anonymous questionnaire distributed to HCWs; ii.) semi-structured interviews with facility managers; and iii.) direct observations made by researchers during site visits.

**Box 1. Guidelines targeted for evaluation.**

**Managerial controls**
Establish and strengthen infection control (IC) committees
Provide training of all staff in IC
Write and disseminate IC standard operating protocols

**Administrative controls**
Screen all patients entering the facility for cough
Promptly triage of presumptive TB patients
Educate patients on cough hygiene
Encourage hospitalized TB patients to spend daylight hours outside
Restrict visitors to TB wards
Physically separate HIV-positive patients from those with known or suspected infectious tuberculosis

**Environmental controls**
Keep windows open when possible
Use fans in enclosed spaces
Provide coughing patients with surgical masks

**Personal protective equipment**
Provide HCWs with N95 respirators

**Access to care for health workers**
Access to confidential HTC, ART, and IPT
Access to TB screening [23]

### Health care worker questionnaire

A short questionnaire was developed and piloted in English, the official language of Malawi (S1 Appendix). At each facility, the purpose of the study was described and questionnaires were distributed during morning report. All facility staff were invited to participate. Formal informed consent was waived for the questionnaire due to the operational nature of the study and the minimal risk involved, however the introduction included detailed information about the voluntary nature of the study, and the absence of punitive action for opting out.

### Manager interviews and direct observations

A tool was developed and piloted in English to guide semi-structured interviews with facility managers (S2 Appendix). Interviews were conducted with at least two managers at each facility using a standardized tool. Facility managers were defined as HCWs in formal leadership positions whose responsibilities encompassed IC. The selection of interviewees was guided by either the District Health Officer or the senior leadership of the facility. Verbal informed consent was obtained from interviewees. Two researchers jointly conducted the interviews, completed separate forms for each interview, and reconciled differences based on shared agreement using the audio recording if necessary. No incentive was offered to interviewees.

### Direct observations

Study staff recorded observations using a standardized checklist on a pre-selected group of IC measures (S3 Appendix). These observations detailed the presence and operation of ventilation systems, whether windows were open, and whether IC protocols were in place at clinic spaces, among other items. Site visits were part of routinely scheduled Tingathe clinical mentorship visits, allowing study staff to observe IC implementation under routine settings (versus specific preparation for an IPC assessment).

### Background facility data

Health facility catchment area population was abstracted from semi-permanent data,[24] attendance figures from the Health Management Information System and Integrated HIV Program Reports published by the Malawi MOH [25,26].

### Analysis

Univariate statistics were used to describe proportions, medians, and interquartile ranges. Summary variables for Likert scores were generated, with agreement defined as a response of “Agree” (4/5) or “Strongly Agree” (5/5). Two-tailed student’s t-test were used to compare continuous variables. Analysis was conducted using STATA Special Edition 14.1 (Stat-Corp LP, College Station, TX, USA).

### Ethics

This study was approved by the Malawi National Health Science Research Committee and the Baylor College of Medicine Institutional Review Board.

## Results

Median facility catchment area population at surveyed facilities was 52,805 (Table 1). Three facilities were in urban areas, and 14 were rural.

**Table 1:** Characteristics of survey sites.

Characteristic	Site type		
	Hospital (N = 6)	Health center (N = 11)	Total (N = 17)
Median facility catchment area population (IQR)	56,778 (38,404–65,897)	45,132 (27,406–56,561)	52,805 (29,604–60,565)
Median annual OPD attendance (IQR)*	89,317 (61,352–166,870)	32,784 (30,905–95,669)	54,256 (30,975–111,874)
Median annual number of admissions (IQR)*	7,660 (6,330–18,564)	926 (735–1074)	1,074 (783–6,330)
Median number of patients active on ART (IQR)	1263 (1070–4770)	541 (381–2202)	893 (487–2,202)
Median annual number of TB cases (IQR)†	102 (52–174)	11 (4.5–87)	50 (7–153)
Location—n (%)			
Urban	0 (0)	3 (27)	3 (18)
Rural	6 (100)	8 (73)	14 (82)

*Not unique patients

^†^3 Health centers missing data

### Respondent characteristics

592 HCWs from seventeen facilities completed anonymous questionnaires. The median age of respondents was 31 years (IQR 26–37) and the most common cadres were health surveillance assistants (19.9%), nurses (16.2%), and community health workers (15.0%; Table 2). Interviews were conducted with thirty-five managers. The median age of interviewees was 38 years (IQR 35–43) years and the median years of management experience was 5 (IQR 3–10).

**Table 2:** Characteristics of HCW respondents (N = 592).

Characteristic	
Age in years—median (IQR)	31 (26–37)
Years working in health care—median (IQR)	6 (3–10)
**Gender—n (%)**	
Male	267 (45.1)
Female	297 (50.2)
No response	28 (4.7)
**Position—n (%)**	
Health surveillance assistant	118 (19.9)
Nurse	96 (16.2)
Community health worker	89 (15.0)
Student	73 (12.3)
Cleaner	68 (11.5)
Medical assistant	30 (5.1)
Clinical officer	24 (4.1)
Clerk	19 (3.2)
Environmental health officer	6 (1.0)
Other*	40 (6.8)
No response	29 (4.9)

*Other includes: hospital attendants, lab technicians, nutritionists, security guards, volunteers, and messengers

### Managerial activities

**Fig 1:**
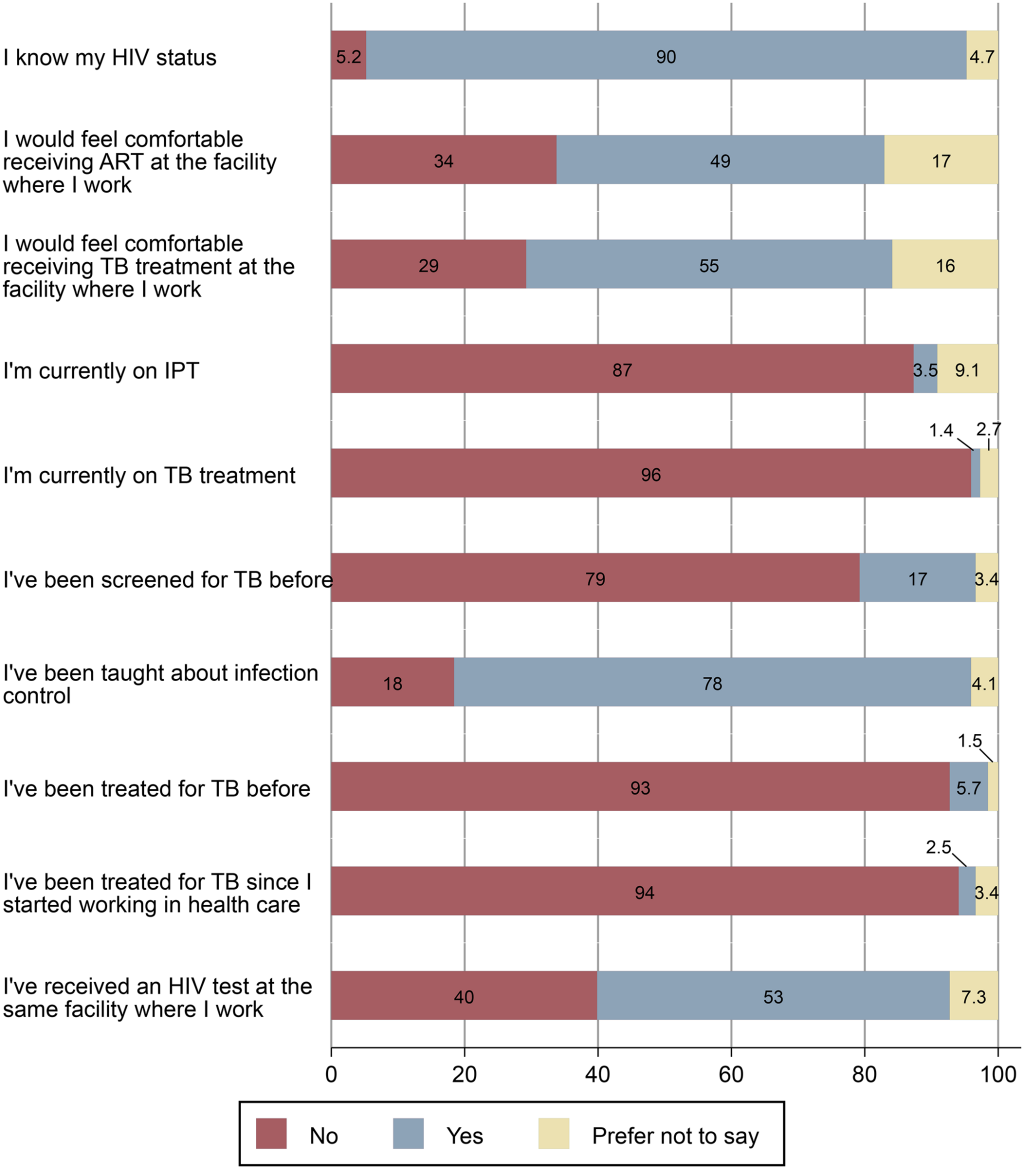
Pooled health worker responses. Numbers correspond with percent of respondents.

Of the 592 HCW respondents, 78% reported ever receiving IPC training (Fig 1); of these, less than half provided an estimated date for their last training (median years elapsed = 1.7, IQR 0.5–3.1), and 66 (11% of all respondents) reported IPC training within the last year. Of seventeen facilities, 10 (59%) had separate waiting areas for TB and ART patients.

### Administrative controls

**Fig 2:**
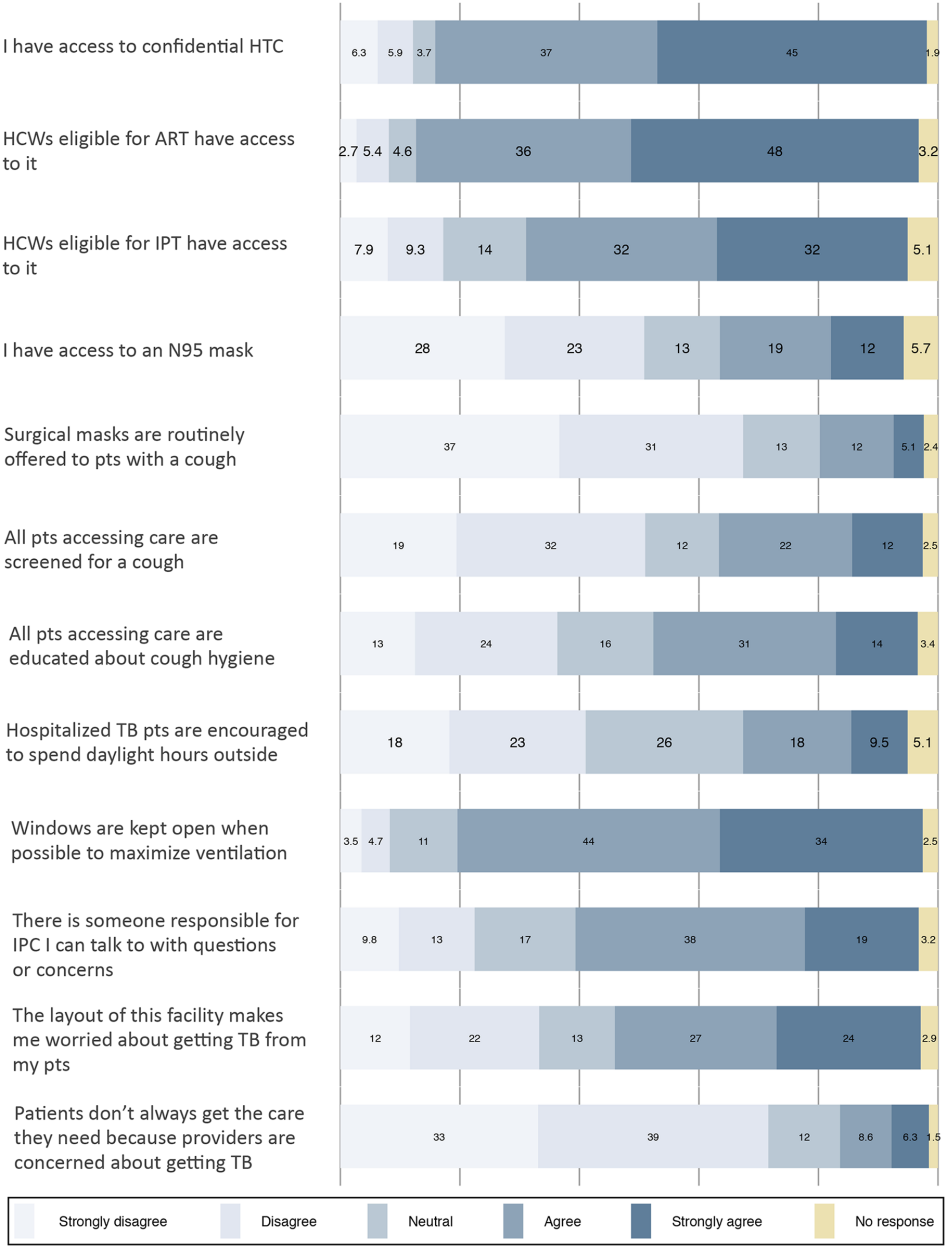
Pooled health worker responses. Numbers correspond with percent of respondents.

Of the 592 HCW respondents, 34% agreed that all patients entering the facility were screened for a cough (Fig 2). Of 592 HCW respondents, 236 (40%) provided cough as a TB symptom, and 48 (8%) correctly named the four most common symptoms of active TB in adults (Table 3). Of 33 managers reporting that their facility conducts symptom screening for TB, only 7 (21%) could name the appropriate screening questions for adults and 1 (3%) named them for children. Of 33 managers indicating their facility conducts symptom screening, half reported this is done at ART clinic. All managers reported use of sputum smear microscopy to evaluate presumptive TB patients, and most (83%) perform the test on-site. Among managers, 43% reported use of GeneXpert.

**Table 3:** Answers from health workers and managers to questions regarding TB screening in adults and children.

Measure	n (%)
Health workers providing 4 most common symptoms of active TB in adults (N = 592)	
Zero	44 (7)
One	134 (23)
Two	208 (35)
Three	158 (27)
Four	48 (8)
Managers providing 4 TB screening questions used in adults (N = 33)	
Zero	2 (6)
One	2 (6)
Two	9 (27)
Three	13 (39)
Four	7 (21)
Managers providing 4 TB screening questions used in children (N = 33)	
Zero	4 (12)
One	9 (27)
Two	9 (27)
Three	10 (30)
Four	1 (3)

### Environmental controls and personal protective equipment

Of 592 HCW respondents, only 182 (31%) reported access to N95 respirators and no managers reported providing HCWs with N95 respirators. No sites were observed to have fans, ultraviolet lights, or air filters in use (Table 4).

**Table 4:** Number of facilities implementing IPC guidelines according to data source (N = 17).

Measure—n (%)	HCW survey^1^	Manager response^2^	Observation
	n (%)	n (%)	n (%)
*Administrative controls*			
Patients are screened for a cough	3 (18)	16 (94)	—
Patients are educated about cough hygiene	7 (41)	6 (35)	—
Hospitalized TB patients are encouraged to spend daylight hours outside	1 (6)	0 (0)	—
*Environmental controls*			
Windows kept open when possible	16 (94)	12 (71)	16 (94)
HCWs have access to N95 respirators	2 (12)	0 (0)	0 (0)
Surgical masks are offered to patients with a cough	0 (0)	0 (0)	0 (0)
Fans used	—	1 (6)	0 (0)
Ultraviolet lights used	—	0 (0)	0 (0)
*Access to care for health workers*			
Policy for regular screening of health workers*	0 (0)	1 (6)	—

*For HCW survey, facilities were counted if a majority of respondents had been screened within the previous 365 days

—Item was not measured in corresponding data source

### HCW access to care

Most HCWs reported they have access to confidential HTC (82%), ART (84%), and IPT (64%). Roughly half of the respondents indicated they would feel comfortable receiving ART (49%) or TB treatment (55%) at the same facility where they work. Of note, these two questions had the highest non-response rate (17% and 16%, respectively). Participants who would feel comfortable receiving ART at their place of work were older (33.8 vs 31.1 years, p = 0.0013) and had worked in health care longer (9.0 vs 7.0 years, p = 0.0068). Almost all of the 592 HCW respondents (90%) reported knowledge of their HIV status, however only 53% received an HIV test at the same facility where they work.

Among managers, 20% reported a policy of regular TB screening for HCWs. Of HCWs, 17.4% of HCWs surveyed reported having ever been screened for TB. Of these, half could approximate the date of their last screen (median years elapsed = 2.5, IQR 1.1–6.4). Among all respondents, 2.4% had been screened in the previous year. Thirty-four (5.7%) respondents reported previous TB treatment. Of the 34 respondents reporting previous TB treatment, 12 (35%) indicated they received it at the same facility where they work. Of all 592 HCW respondents, 8 (1.4%) reported current TB treatment and 21 (3.5%) reported current IPT. Over half of respondents (51%) indicated the layout of their facility makes them concerned about getting TB from patients.

## Discussion

This study is the first in over a decade to document the current state of IC guidelines in public facilities in Malawi. Further, it is the first in the era of widespread ART availability and task shifting to characterize access to care for HCWs. Our findings suggest that IC guideline implementation is inadequate, and bring to light issues surrounding HCW access to care.

Our findings raise concern over the state of routine symptom screening for TB. Most anonymous respondents did not agree that screening is routine. Further, most of the manager respondents were unable to list the screening questions for TB in adults, and only one provided the correct questions for children. Together, these findings suggest that TB screening is not routinely performed, and that when it does occur, is likely undercut by knowledge gaps.

Screening for TB is an upstream element of IC—almost all other components require an understanding of who is potentially infectious, and who is not. This understanding is only possible through robust, routine screening protocols. If screening is impaired, downstream targeted interventions—such as isolation of infectious patients and use of N95 respirators—are of little use, placing HCWs and other patients at risk. The shockingly high incidence of TB identified on autopsy studies among patients who were not suspected of having TB highlights the importance of this initial step [27,28].

Screening as part of a larger IC strategy has been described in the acronym FAST: Find cases Actively, Separate safely, and Treat effectively based on rapid drug susceptibility testing (DST)[29]. By coupling robust screening with improved diagnostics, this approach holds potential to halt the chain of transmission. With the growing availability of the GeneXpert rapid molecular test, the infrastructure is in place to more effectively evaluate individuals who screen positive. While significant, investments made now towards rapid and accurate case identification could reduce transmission and slow the development of multidrug-resistant TB, which confers exponentially greater costs per case transmitted.

Our findings suggest suboptimal implementation of several other IC measures. Of note, no fans or germicidal ultraviolet lights (GUVs) were observed in use. While windows and doors were kept open, relying entirely on simple natural ventilation grows more difficult as the size and complexity of a facility grows [30]. Fans offer the simplest solution to improve ventilation in smaller facilities with direct exhaust paths to the outside air. For more complex facilities, Malawi guidelines recommend the use of GUVs, however uptake has been limited by their high initial costs, maintenance requirements, and need for a reliable source of power [23]. Reconsideration of GUVs may soon be warranted, given the development of cheaper LED fixtures that can run on batteries and solar power. This is especially pertinent in large facilities where the cost per incremental air change is nine times lower for GUV compared to mechanical ventilation [31].

Our findings add to a limited body of evidence pointing to barriers for HCWs in accessing diagnostic and treatment services for HIV and TB [15–17,32]. The data is troubling: only one site reported a policy for regular HCW screening for TB, fewer than one in five HCWs reported ever being screened for TB, and only 2% of all HCWs had been screened in the last year. While most HCWs reported knowing their HIV status, a high proportion were not tested at their site of employment, and only half reported they would feel comfortable receiving ART at the facility where they work. These results are in line with other work suggesting that perceived lack of confidentiality at their facility of employment is a significant barrier for HCWs’ uptake of HIV testing services [15].

Preventive services for HCWs should be commensurate with the incremental risk they face in their routine tasks. This is particularly relevant in Malawi, where the health workforce is already stretched thin [33]. Access to HIV testing and ART—the most effective prevention measure for PLHIV regardless of profession—is critical, as is ensuring regular screening of HCWs for TB [34]. The consequences of inaction are well-documented: increased morbidity among HCWs, and also their patients, who would face increased exposure from their care providers [14].

There are several important limitations of this study. Most measurements relied on self-reporting, introducing the possibility of social desirability bias. We attempted to minimize bias by drawing on a large sample of anonymous HCW responses, and by including objective observations in our analysis. Our use of a convenience sample introduces the potential that our sites are not representative of Malawi as a whole.

## Conclusions

As the numbers of persons on ART increase, it is critical to ensure health facilities are not conferring increased risk to individuals accessing care. Current implementation of IC guidelines appears to be suboptimal, and barriers to care persist for HCWs. Improvements are needed to protect patients and HCWs in health settings. Robust implementation of routine symptom screening to rapidly identify presumptive TB patients and access to confidential HIV/TB-related services for HCWs is paramount to reduce the risk of nosocomial TB transmission and promote a safer health care setting.

## Supporting information

S1 Appendix Health care worker questionnaire. (PDF)

S2 Appendix Manager interview guide. (PDF)

S3 Appendix Observation data recording tool. (PDF)

S1 Data Complete study dataset, separated into health worker responses (ipc_hcw.dta), manager responses (ipc_manager.dta), and facility observations (ipc_obs.dta). (ZIP)
