# Supplementary material for: Assessing infection control practices to protect health care workers and patients in Malawi from nosocomial transmission of Mycobacterium tuberculosis
Source: PLoS One. 2017 Dec 6;12(12):e0189140. doi: 10.1371/journal.pone.0189140 (PMC5718482; doi:10.1371/journal.pone.0189140)
Supplement: S1 Appendix — (PDF) [file pone.0189140.s001.pdf]

Date:____/____/____ Site: ______________________________ #2-__________

Tingathe Infection Prevention and Control Survey

Thank you for offering to share your thoughts. Your voluntary feedback will remain **anonymous** and is important to strengthen infection control practices that protect you, your colleagues, and your patients. Surveys will be totaled by site for reporting purposes and to identify areas for improvement. **This is NOT an assessment of individual performance.**

If you have feedback, questions, or concerns for the study team, please contact your Tingathe mentor.

1	What is your age?	
2	What is your gender?	☐Male ☐Female ☐Prefer not to say
3	What is your position? *(Check all that apply)* ☐Other:_____________	☐Nurse ☐Clinical officer ☐Manager ☐Clerk ☐Student ☐Tingathe CHW ☐Medical doctor ☐HSA ☐Medical assistant ☐Prefer not to say ☐Cleaner ☐EHO
4	How many years have you been working in health care?	

	**Please check one box best describing your level of agreement with each statement** ***At my facility…***	**Strongly disagree**	**Disagree**	**Neutral**	**Agree**	**Strongly agree**
5	I have access to confidential HTC					
6	Health care workers eligible for **antiretroviral therapy (ART)** have access to it					
7	Health care workers eligible for **isoniazid preventive therapy (IPT)** have access to it					
8	I have access to an N95 face mask if I want one					
9	Surgical masks are routinely offered to patients with a cough					
10	All patients accessing care are screened for cough					
11	All patients accessing care are educated about cough hygiene					
12	Hospitalized TB patients are encouraged to spend daylight hours outside					
13	Windows are kept open when possible to maximize ventilation					
14	There is someone responsible for infection control I can talk to with questions or concerns					
15	The physical layout of this facility makes me worried about getting TB from my patients					
16	Patients don't always get the services they need because providers are concerned about getting TB					

17	I know my HIV status	☐Yes ☐No ☐Prefer not to say
18	I've received an HIV test at the same facility where I work	☐Yes ☐No ☐Prefer not to say
19	I've been treated for TB before	☐Yes ☐No ☐Prefer not to say
20	I've been treated for TB since I started working in health care	☐Yes ☐No ☐Prefer not to say
20.1	↳*If yes:* I received treatment at the facility where I work	☐Yes ☐No ☐Prefer not to say ☐Not applicable
21	I'm currently on TB treatment	☐Yes ☐No ☐Prefer not to say
22	I'm currently on IPT	☐Yes ☐No ☐Prefer not to say
23	I would feel comfortable receiving **TB treatment** at the facility where I work	☐Yes ☐No ☐Prefer not to say
24	I would feel comfortable receiving **ART** at the facility where I work	☐Yes ☐No ☐Prefer not to say
25	Have you ever been screened for TB?	☐Yes ☐No ☐Prefer not to say
26	When were you last screened for TB? (mm/yy)	____/____ ☐Never ☐Don't remember
27	Have you ever been taught about infection control?	☐Yes ☐No ☐Prefer not to say
28	When was the last time you were taught about infection control? (mm/yy)	____/____ ☐Never ☐Don't remember
29	What are the four most common signs and symptoms of active TB disease in adults?	1.______________ 2.________________<br>3.______________ 4.________________
30	In the next year, what do you think your risk is of becoming infected with TB? (*Please choose one answer)*<br>0% ☐ *Definitely won't get TB* — 25% ☐ *Probably won't get TB* — 50% ☐ *Might get TB* — 75% ☐ *Probably will get TB* — 100% ☐ *Definitely will get TB*	

This concludes the survey. Thank you very much for your participation.
