# Supplementary material for: Assessing infection control practices to protect health care workers and patients in Malawi from nosocomial transmission of Mycobacterium tuberculosis
Source: PLoS One. 2017 Dec 6;12(12):e0189140. doi: 10.1371/journal.pone.0189140 (PMC5718482; doi:10.1371/journal.pone.0189140)
Supplement: S2 Appendix — (PDF) [file pone.0189140.s002.pdf]

Tingathe TB - Infection Control Survey of Facility Managers

Date (dd/mm/yy):____/____/____ Site:_________________ **#:1-__________________**

Respondent name:___________________ Dept:________________ Length of time as manager:_______ yrs

Age:_____ Title:________________ Name of person filling form:____________________ *v9, 8/9/14*

Section I - Administrative controls

#	Question	Written Response	Jump to
1	Does your facility conduct patient education sessions?	☐No--<br>☐Yes	→Q2
1.1	What topics are covered with respect to TB?<br>*(Check all that apply)*	☐Cough hygiene<br>☐TB transmission/prevention-----------------------------------<br>☐HIV & TB<br>☐TB diagnosis<br>☐Symptoms of TB<br>☐Treatment of TB<br>☐None of the above<br>☐Other:_____________________________________	→probe
1.2	Who is targeted for patient education?<br>*(Check all that apply)*	☐Patients accessing TB services<br>☐Patients accessing HIV services<br>☐Patients accessing NRU services<br>☐Everyone entering facility<br>☐TB suspects<br>☐None of the above<br>☐Other:_____________________________________	
2	When patients come to the OPD, how do you decide who to see first?<br>*(Check all that apply)*	☐No prioritization<br>☐Critically ill patients<br>☐All patients with cough<br>☐All TB suspects<br>☐Other:_____________________________________	
3	What preventive measures are in place for patients with a cough **suspected of having TB?**<br>*(Check all that apply)*	☐Masks/tissues offered to patients<br>☐Face masks for health care workers (generic)<br>☐Face masks for health care workers (N-95)<br>☐Triage and isolation<br>☐Cough etiquette education<br>☐None of the above	
4	What preventive measures are in place for hospitalized patients **receiving TB treatment?**<br>*(Check all that apply)*	☐Masks/tissues offered to patients<br>☐Face masks for health care workers<br>☐Triage and isolation<br>☐Placed in part of ward with good ventilation<br>☐Encouraged to spend daylight hours outside<br>☐None of the above<br>☐Other:__________________________________<br>☐No hospitalized patients at site--------------------------------	→Q7
5	Is there a policy addressing visitor's access to hospitalized patients being treated for TB?	☐No policy minimizing visitors<br>☐Policy minimizing visitors	

5.1	What groups are specifically restricted from entering TB ward?<br>*(Check all that apply)*	☐Children <5 years<br>☐Pregnant women<br>☐No groups restricted<br>☐Other:___________________________________	
6	Is there a policy outlining where health care workers living with HIV should or should not work?	☐No<br>☐Yes, isolates HIV+ health care workers from TB suspects/patients<br>☐Yes, but doesn't isolate HIV+ health care workers	
7	Are there standard operating procedures for infection prevention and control?	☐No<br>☐Yes	
8	Is there an infection control committee?	☐No--<br>☐Yes	→Q10
8.1	Is there a point person assigned for this committee?	☐No<br>☐Yes	
8.2	Name:		
8.3	Cadre:		
8.4	Contact:		
		**Section II - Case finding**	
9	Is active symptom screening conducted for TB?	☐No--<br>☐Yes	→Q11
9.1	Where is symptom screening conducted for TB?<br>*(Check all that apply)*	☐ART/HCC<br>☐ANC<br>☐Malnutrition<br>☐General inpatient wards<br>☐OPD<br>☐HTC<br>☐Public Health<br>☐Other:______________________________	
9.2	Which groups of people coming to this facility get screened for symptoms of TB?<br>*(Check all that apply)*	☐Patients<br>☐Clients<br>☐Guardians	
9.3	What questions are asked to screen **adults** for TB?<br>*(Check all that apply)*	☐Cough<br>☐Fever<br>☐Weight loss<br>☐Night sweats<br>☐Other:____________________________<br>☐Other:____________________________	

9.4	What questions are asked to screen **children** for TB?<br>*(Check all that apply)*	☐Cough<br>☐Fever<br>☐Poor weight gain/failure to thrive<br>☐Home TB contact<br><br>☐Other:____________________________<br><br>☐Other:____________________________	
9.5	How many patients have been screened for symptoms of TB in the last month?		
9.6	How many patients have screened positive for symptoms of TB in the last month?		
10	What diagnostics are routinely used to evaluate TB suspects?<br>*(Check all that apply)*	☐Sputum smear-------→☐At this facility ☐Sent to another facility<br>☐GeneXpert------------→☐At this facility ☐Sent to another facility<br>☐Culture----------------→☐At this facility ☐Sent to another facility<br>☐Chest X-Ray----------→☐At this facility ☐Sent to another facility<br>☐None of the above	
11	Is there a policy for regular screening of health care workers for TB?	☐No<br>☐Yes	
12	How many health care workers have been treated for active TB in the last year?		
**Section III - Environmental Controls**			
13	Are there single-patient isolation rooms?	☐No<br>☐Yes	
14	Are fans in place and routinely used?	☐No<br>☐Yes	
15	Are UV lights in place and routinely used?	☐No<br>☐Yes	
16	Are air filters in place and routinely used?	☐No<br>☐Yes	
17	What policies are in place to maximize natural ventilation?	☐None/other<br>☐Windows/doors kept open	
