# Supplementary material for: Assessing infection control practices to protect health care workers and patients in Malawi from nosocomial transmission of Mycobacterium tuberculosis
Source: PLoS One. 2017 Dec 6;12(12):e0189140. doi: 10.1371/journal.pone.0189140 (PMC5718482; doi:10.1371/journal.pone.0189140)
Supplement: S3 Appendix — (PDF) [file pone.0189140.s003.pdf]

Tingathe TB - Infection Control Observations

Section I - Administrative controls

Date (dd/mm/yy):____/____/____ Site:________________ **#3-__________**

Name of person filling form:________________________ *v06 8/9/14*

#	Question	Written Response
1	UV lights observed and switched on	☐No<br>☐Yes
2	Filtration units observed and switched on	☐No<br>☐Yes
3	Fans observed and switched on	☐No<br>☐Yes
4	At ART clinic, were the windows open?	☐No windows open<br>☐Some windows open<br>☐All windows open
5	At the TB office, were the windows open?	☐No windows open<br>☐Some windows open<br>☐All windows open
6	At malnutrition clinic, were the windows open?	☐No windows open<br>☐Some windows open<br>☐All windows open<br>☐No malnutrition clinic at site
7	Copy of infection control standard operating procedures present in areas where patients are seen	☐No<br>☐Yes
8	Waiting area for HIV services physically separated from waiting area for TB patients	☐No<br>☐Yes
9	Patients at ART/HCC observed wearing a mask	☐No<br>☐Yes

Comments:
